# Validity and reproducibility of a novel method for time-course evaluation of diet-induced thermogenesis in a respiratory chamber

**DOI:** 10.14814/phy2.12410

**Published:** 2015-05-27

**Authors:** Chiyoko Usui, Takafumi Ando, Kazunori Ohkawara, Rieko Miyake, Yoshitake Oshima, Masanobu Hibi, Sachiko Oishi, Kumpei Tokuyama, Shigeho Tanaka

**Affiliations:** 1 Department of Nutritional Science, National Institute of Health and Nutrition, National Institutes of Biomedical Innovation, Health and NutritionTokyo, Japan; 2 Japan Society for the Promotion of ScienceTokyo, Japan; 3 Department of Communication, Tokyo Woman's Christian UniversityTokyo, Japan; 4 Faculty of Informatics and Engineering, University of Electro-CommunicationsTokyo, Japan; 5 Faculty of Service Industries, University of Marketing and Distribution SciencesKobe, Japan; 6 Health Care Food Research Laboratories, Kao CorporationTokyo, Japan; 7 Institute of Health and Sport Sciences, University of TsukubaTsukuba, Japan

**Keywords:** Diet-induced thermogenesis, reproducibility, respiratory chamber, triaxial accelerometry, validity

## Abstract

We developed a novel method for computing diet-induced thermogenesis (DIT) in a respiratory chamber and evaluated the validity and reproducibility of the method. We hypothesized that DIT may be calculated as the difference between postprandial energy expenditure (EE) and estimated EE (sum of basal metabolic rate and physical activity (PA)-related EE). The estimated EE was derived from the regression equation between EE from respiration and PA intensity in the fasting state. It may be possible to evaluate the time course of DIT using this novel technique. In a validity study, we examined whether DIT became zero (theoretical value) for 6 h of fasting in 11 subjects. The mean value of DIT calculated by the novel and traditional methods was 22.4 ± 13.4 and 3.4 ± 31.8 kcal/6 h, respectively. In the reproducibility study, 15 adult subjects lived in the respiratory chamber for over 24 h on two occasions. The DIT over 15 h of postprandial wake time was calculated. There were no significant differences in the mean values of DIT between the two test days. The within-subject day-to-day coefficient of variation for calculated DIT with the novel and traditional methods was approximately 35% and 25%, respectively. The novel method did not have superior reproducibility compared with that of the traditional method. However when comparing the smaller variation in the fasting state than the theoretical value (zero), the novel method may be better for evaluating interindividual differences in DIT than the traditional method and also has the ability to evaluate the time-course.

## Introduction

Diet-induced thermogenesis (DIT) is commonly defined as the increment in energy expenditure (EE) above the fasting state that is associated with digestion, absorption, and storage of food. DIT accounts for 5–15% of total daily EE (TEE) (Schutz et al. 1984; Van Zant 1992; Westerterp 2004) and is a unique physiological phenomenon that varies in individuals due to the effects of meal characteristics (size, composition, timing, and frequency) (de Groot et al. 1989; Weststrate et al. 1989; Segal et al. 1990; Verboeket-van de Venne et al. 1993) and physiological factors (subject's genetic background, age, physical fitness, and insulin sensitivity) (Schutz et al. 1984; D'Alessio et al. 1988; Segal et al. 1990, 1990, 1992; Minghelli et al. 1991; Tataranni et al. 1995). The predicted values of DIT also show relatively large errors due to methodological problems (indirect calorimetry equipment, interfering environmental factors, duration of measurements, and the position of the participants) (Ravussin et al. 1986; Minghelli et al. 1991; Weststrate 1993; Tataranni et al. 1995; Granata and Brandon 2002; Westerterp 2004). However, DIT data still has great potential to assist with further developments in physiology and medicine. Although the major factors involved in DIT have been studied widely, many issues persist in the literature and research.

Diet-induced thermogenesis is generally measured using ventilated hood systems (Weststrate et al. 1989; Segal et al. 1992; Weststrate 1993) or respiratory chambers (Schutz et al. 1984; Ravussin et al. 1986; Tataranni et al. 1995). The use of a respiratory chamber to measure DIT has the advantage of simultaneously assessing TEE and its components and substrate utilization under simulated real-life conditions over an entire day. The first attempts to assess DIT in a respiratory chamber were performed by Schutz et al. (1984). These researchers calculated DIT over a longer period in the chamber using the intercept of the regression line of EE versus physical activity (PA) frequency during the postprandial state, assessed using a radar system. However, several studies have demonstrated numerous flaws and concerns regarding this method (Ravussin et al. 1986; Tataranni et al. 1995; Marino et al. 2002; Kumahara et al. 2011). First, Tataranni et al. (1995) reported that the measurement of DIT in a respiratory chamber using Schutz's method was not ideal due to poor reproducibility compared with that of DIT using a ventilated hood system (Segal et al. 1992; Weststrate 1993). They suggested that the reasons for poor reproducibility were likely to be related to large variability in the terms used to calculate DIT, that is, basal metabolic rate (BMR) and the intercept of the regression line between EE and PA. In addition, they showed that DIT calculated using Schutz's method was underestimated compared to DIT calculated as the difference in 24 h EE when the subjects were fed or fasted. The slope and intercept of the regression line in Schutz's method can also be affected by the timing of high-intensity PA in the chamber. Therefore, Schutz's paper tacitly acknowledged that the method was weak for assessing the time course because of the heterogeneous nature of the variance in the relationship between EE and PA during the postprandial state. In fact, despite adjusting for PA-related EE (PAEE) in a chamber, Schutz's technique cannot determine the time course of DIT, because it assesses average DIT over a longer period. Finally, previous studies also reported (Ravussin et al. 1986; Tataranni et al. 1995) that an index of PA measured using a radar system provided insufficient information for assumptions on the relationship between the magnitude of PA and EE, and led to inaccurate calculation of DIT in a chamber. Kumahara et al. (2011) reported that DIT calculated by the traditional Schutz's method using data of PA from a radar system and an accelerometer that measured the duration and intensity of PA was able to achieve consistent and reliable results and also assess interindividual variability. Our published data indicates that the average intensity of most spontaneous PA, with the exception of special activities such as exercise and housecleaning, when expressed as synthetic acceleration (mG) is <30 mG under free-living conditions in the chamber (Ohkawara et al. 2011). We have reported previously that a triaxial accelerometer with high sensitivity can accurately estimate the intensity of even sedentary and household activities (Oshima et al. 2010; Ohkawara et al. 2011). Therefore, the intensity of PA assessed using a triaxial accelerometer can help to track PAEE in the chamber.

In order to improve the low reproducibility, accuracy, and assessment of the time course, we hypothesized that DIT may be calculated as the difference between measured postprandial EE and estimated EE (i.e., the sum of BMR and PAEE calculated using the regression equation between EE from respiration and monitoring body movement in the fasting state). The novel technique also takes into account the relationship between PAEE evaluated using respiration and acceleration in the fasting state, and might be able to evaluate the time course and magnitude of each energy component, such as BMR, DIT, and PAEE. In addition, we compared whether or not acceleration is better for determining DIT. In this study, we attempted to develop a novel technique for computing DIT in the respiratory chamber and to assess the validity and reproducibility of this technique.

## Methods

The methodology used is described in two parts. *Part 1* describes the validity study on 11 subjects, whereas *part 2* describes the reproducibility study on 15 subjects. Five subjects (3 males and 2 females) participated in both the validation and reproducibility studies. The studies were approved by the Ethical Committee of the National Institute of Health and Nutrition (NIHN). All the subjects received a verbal and written description of the study and gave their informed consent to participate prior to testing.

### Methods for evaluating DIT using an indirect human calorimeter

#### Method 1 (novel NIHN method)

We developed a novel evaluation method for computing DIT in a respiratory chamber at the NIHN (Tokyo, Japan). The subjects performed four activities under fasting conditions in the morning while wearing a triaxial accelerometer on the right side of their waist in a respiratory chamber. The selected activities were as follows: resting in the sitting position (i.e., resting metabolic rate (RMR)), personal computer work, doing a jigsaw puzzle, and folding the laundry. These activities were chosen as representative activities of low-intensity physical activity (PA) in a respiratory chamber, based on our observations of a preliminary study of the activity records of other subjects. All the activities were performed for 20 min, respectively (total 80 min). The energy expenditure (EE) and triaxial accelerations or percentage of activity measured by a motion-detecting system (infrared sensor output) in the subjects during each activity were obtained under the steady state. The period of measurement was from 2 min after starting the activity to 1 min before stopping the activity. The estimated EE (EEe, including BMR and PAEE, except DIT) during four activities in the fasting state was calculated using the regression equation of EEs and synthetic accelerations (*Method 1-a: M1a*) or infrared sensor output (*Method 1-b: M1b*).



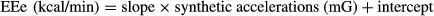
1a



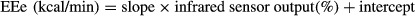
1b

Diet-induced thermogenesis was therefore determined by subtracting EEe from measured EE in the postprandial state.



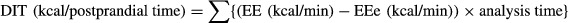
2

#### Method 2 (Schutz's method)

The DIT was calculated as proposed by Schutz et al. (1984). EE was plotted against synthetic accelerations (*Method 2-a: M2a*) or infrared sensor output (*Method 2-b: M2b*), both averaged over 30 min periods during the postprandial state, except for sleep time. The intercept of the regression line, represents EE in the inactive state (i.e., RMR) and consists of BMR and DIT. DIT was determined by subtracting BMR from RMR.



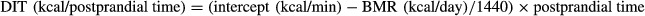
3

Because we did not measure BMR in this study, we calculated BMR using the following predicted equation.



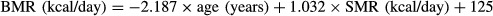
4(SMR: sleeping metabolic rate, *R*^2 ^= 0.877, SEE = 91.8).

This equation was developed to predict BMR by stepwise multiple regression analysis of data from our previous study on 70 males and 65 females (age: 20.7–74.9 years, BMI: 16.3–36.4 kg/m^2^; unpublished our data). SMR was defined as the minimum EE for 3 consecutive hours of sleep.

### Indirect human calorimeter

This study used two open-circuit respiratory chambers to evaluate EE. The performance and details of the human calorimeter have been described previously (Futami et al. 2003; Ganpule et al. 2007). Briefly, the respiratory chambers were airtight rooms (20,000 and 15,000 L, respectively) equipped with a bed, desk, chair, TV with a DVD recorder and HDD, CD player, telephone, toilet, sink, and cycle ergometer. The temperature and relative humidity in the rooms were controlled to be comfortable for the subject at 25°C and 55%, respectively. The oxygen and carbon dioxide concentrations of the air supply and exhaust were measured by mass spectrometry. In all the experiments, the mass spectrometer (ARCO-1000A-CH; Arco System, Kashiwa, Japan) was calibrated initially using a certified gas mixture and atmospheric air. The inlet air supply from outside, expired air from the chamber, and atmospheric air in the gas bomb were analyzed every 3 sec during the experiment (1 set = total 12 sec). Mass spectrometry was calibrated every 12 sec based on atmospheric air. The flow rate exhausted from the chamber was measured by pneumotachography (FLB1; Arco System, Kashiwa, Japan). The flow meter was calibrated before each experiment, and the flow rate maintained at ˜60 L/min. Oxygen uptake (VO_2_) and carbon dioxide production (VCO_2_) were determined by the flow rate of exhaust from the chamber, and the concentrations of the inlet and outlet air of the chamber, respectively (Futami et al. 2003). EE was estimated from VO_2_ and VCO_2_ using Weir's equation (Weir 1949). The accuracy and precision of our human calorimeter for measuring EE using the alcohol combustion test was 99.8 ± 0.5% (mean ± standard deviation) over 6 h and 99.4 ± 3.1% over 30 min.

### Triaxial accelerometer and motion-detecting sensor

Three-dimensional accelerations during physical activities were obtained by a triaxial accelerometer (LIS3LV02DQ; ST-Microelectronics, Geneva, Switzerland). The sensor was built into a plastic case without a liquid crystal display and was designed to be clipped to a waist belt (size: 80 × 50 × 20 mm; weight: approximately 60 g including batteries). The anteroposterior (*x*-axis), mediolateral (*y*-axis), and vertical (*z*-axis) acceleration measurements were obtained during each activity at a rate of 32 Hz with 12 bit accuracy. The range of the acceleration data of each axis was ± 6 G, resulting in a resolution of 3 mG. The acceleration data were uploaded to a personal computer. The signals obtained from the triaxial accelerometer were processed in the following way. Each of the three signals from the triaxial accelerometer was passed through a high-pass filter with a cut-off frequency of 0.7 Hz in order to remove the gravitational acceleration component from the signal. We calculated the synthetic acceleration of all three axes using signals after high-pass filtering.

Spontaneous PA was evaluated using a motion-detecting system. The chamber had two independent sensors of the passive infrared type (Matsushita Automation Controls Co, Ltd, AMP2009B01, Tokyo, Japan) that detected movement at speeds > 7 cm/s with a 1 sec epoch. When at least one sensor detected movement, the movement was regarded as positive. The system measured the percentage of time when movement was observed during each minute.

### Part 1: The validity study

#### Subjects

Eleven subjects (6 males and 5 females) participated in this study. All the subjects were adults (≥ 20 years) and did not have chronic diseases that could affect metabolism or daily PA. The descriptive characteristics of the study subjects are presented in Table1.

**Table 1:** Physical characteristics of the subjects in the validity study 1.

	Total (*n* = 11)	Male (*n* = 6)	Female (*n* = 5)
Age (years)	28.3 ± 4.5	27.1 ± 5.0	29.7 ± 4.0
Height (cm)	167.4 ± 7.7	172.9 ± 4.0	160.9 ± 5.5^1^
Body weight (kg)	60.9 ± 10.5	67.3 ± 8.5	53.1 ± 6.9^2^
BMI (kg/m^2^)	21.6 ± 3.0	22.6 ± 3.4	20.5 ± 2.4
Body fat (%)	19.3 ± 6.6	15.8 ± 6.2	23.5 ± 4.7^1^
Fat-free mass (kg)	49.1 ± 8.9	56.3 ± 3.2	40.4 ± 3.8^1^
Fat mass (kg)	11.8 ± 4.6	11.1 ± 5.4	12.6 ± 3.9

BMI, body mass index. The values are expressed as mean ± SD.

1 *P* < 0.05 calculated by Student's *t*-test.

2 *P* < 0.05 calculated by the Mann–Whitney rank sum test.

#### Experimental protocol

The subjects were fitted at the right waist with a triaxial accelerometer and entered the respiratory chamber at 1850. They consumed a standardized meal (802 kcal, 15.3% protein, 21.4% fat, and 63.3% carbohydrate) at 1900 and were then permitted to sleep at 2300. After getting up at 0600 they performed four activities for 20 min each. During the 6 h from 0800 to 1400 when DIT was calculated, the subjects went without meals in the respiratory chamber. While in the chamber, they were free to drink water and move about, but were not allowed to perform intentional physical exercise or take a nap. In the female subjects, the session was performed during the follicular phase of their menstrual cycle. The DITs over 6 h were calculated using four methods in the fasting state.

### Part 2: The reproducibility study

#### Subjects

Fifteen adults (9 males and 6 females) participated in this study. All the subjects were adults (≥ 20 years) and did not have chronic diseases that could affect metabolism or daily PA. The average physical characteristics of the subjects are described in Table2.

**Table 2:** Physical characteristics of the subjects in the reproducibility study

	Total (*n* = 15)	Male (*n* = 9)	Female (*n* = 6)
Age (years)	28.0 ± 6.4	26.7 ± 7.2	30.0 ± 5.1
Height (cm)	166.9 ± 9.0	172.0 ± 3.8	159.1 ± 9.2^1^
Body weight (kg)	61.3 ± 9.9	66.8 ± 5.8	53 ± 9.1^1^
BMI (kg/m^2^)	21.9 ± 2.3	22.6 ± 2.5	20.8 ± 1.6
Body fat (%)	18.2 ± 6.0	14.9 ± 4.9	23.3 ± 3.3^1^
Fat-free mass (kg)	50.2 ± 9.0	56.6 ± 1.8	40.5 ± 5.8^2^
Fat mass (kg)	11.1 ± 4.1	7.7 ± 1.4	12.5 ± 3.7

BMI, body mass index.

Values are expressed as mean ± SD.

1 *P* < 0.05 calculated by Student's *t*-test.

2 *P* < 0.05 calculated by the Mann–Whitney rank sum test.

#### Experimental protocol

The subjects lived in a respiratory chamber for over 24 consecutive hours beginning at 1850. All the subjects participated in two sessions. The two sessions were separated by an average of 4.3 ± 1.4 days (minimum 3 days, maximum 8 days). In the female subjects, the two sessions were performed during the same phase of their menstrual cycle (i.e., follicular or luteal phase). The subjects ingested the same three standardized meals, namely, dinner (1900), breakfast (0850), and lunch (1350), according to their individual estimated energy needs. The composition of the meals, expressed in terms of energy, was 14.4% protein, 25.3% fat, and 60.3% carbohydrate. The subjects were fed to energy balance as estimated from their predictive BMR, calculated using Ganpule's equation (Ganpule et al. 2007), multiplied by a PA level of 1.3. Total energy intake averaged 2008 ± 284 kcal. After 7 h of sleep they performed four activities in the morning for 20 min each in the fasting state. Water was provided ad libitum during free time in the chamber except for meal periods and during specified activities. The subjects were free to move about or lie down in the chamber as they wished, but were not allowed to perform intentional physical exercise or take a nap. The DIT over 15 h after meals (i.e., for 5 h after dinner, breakfast, and lunch, respectively) was calculated using the four methods.

### Anthropometry and body composition

A digital scale was used to measure body weight to the nearest 0.1 kg, whereas the subjects were dressed in light clothing. Barefoot standing height was measured to the nearest 0.1 cm using a wall-mounted stadiometer. Body mass index was calculated as body weight (kg) divided by height squared (m^2^). The percent of body fat was estimated by the bioelectrical impedance method using a HBF-362 (Omron Healthcare Co., Ltd, Kyoto, Japan). Fat mass and fat-free mass were calculated from body weight and percent of body fat.

### Statistical analysis

The results are presented as the mean and standard deviation (SD). All the statistical analyses were carried out using sigma stat 3.5 (Systat Software Inc., San Jose, CA). The differences were considered statistically significant if the *P* value was < 0.05.

Statistical analysis was performed using unpaired Student's *t*-tests for parametric variables and the Mann–Whitney rank sum test for nonparametric variables to determine the difference between males and females. The DITs over 6 h in the fasting state were compared with the theoretical value (i.e., zero) using the one-sample *t*-test. The *F*-test and Bonferroni method for adjusting *P* values were used to measure the homogeneity of variance between values obtained by the four methods used to calculate DIT. Absolute reproducibility refers to a comparison between the group means for a given variable between *days 1* and *2* of the experiment. Paired Student's *t*-tests were used for these comparisons. Relative reproducibility refers to the concordance of a given subject's measurements between *days 1* and *2* assessed by both linear regression analysis and Pearson's correlation coefficient (*r*_*i*_). Bland–Altman plots were also used to graphically show the variability in individual error scores (Bland and Altman 1986). The coefficient of variation (CV) was calculated for the 15 h mean as described below (Flint et al. 2000):

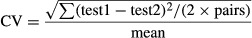


## Results

### Validity study

Table3 presents the DIT over 6 h calculated using the novel and traditional methods. Compared with zero (the theoretical value), the mean values of 6 h DIT calculated using the novel NIHN method (*M1a* and *M1b*) were significantly different (*M1a*: *P* < 0.001, 95% CI (13.43–31.47), *M1b*: *P* < 0.001, 95% CI (25.08–56.16), respectively). In contrast, the mean value of 6 h DIT measured by Schutz's method (*M2a* and *M2b*) was not different (*M2a*: *P* = 0.081, 95% CI [−1.79–26.04], *M2b*: *P* = 0.728, 95% CI [−17.93–24.79], respectively). The mean values of individual correlation coefficients between EEs and synthetic accelerations or infrared sensor outputs observed during the four activities using the NIHN method were 0.837 (range: 0.558–0.999) in *M1a and* 0.757 (0.212–0.985) in *M1b*, respectively. On the other hand, the mean values of individual correlation coefficients between EEs and synthetic accelerations or infrared sensor outputs observed during postprandial measurements (i.e., 1 data/30 min) using Schutz's method were 0.709 (0.403–0.964) in *M2a and* 0.589 (0.099–0.930) in *M2b*, respectively. The calculated DITs of the four subjects in the *M2a* group and six subjects in the *M2b* group were negative values. The estimated BMRs were larger than the y-intercept (i.e., zero activity) in these cases. The mean value of DIT calculated using the novel NIHN method (*M1a*) and the traditional method with a motion-detecting system (*M2b*) was 22.4 kcal/6 h and 3.4 kcal/6 h, respectively. The SD of DIT measured by *M1a* was smaller than that measured by *M2b* (*F* = 5.601, *P* = 0.071 (adjusted by the Bonferroni method)). However, there was no statistically significant difference in the homogeneity of variance between the values.

**Table 3:** Diet-induced thermogenesis over 6 h calculated using the four methods.

Subject	Method			
	M1a	M1b	M2a	M2b
1	39.5	50.5	−13.3	−40.4
2	46.3	74.7	36.9	20.9
3	12.9	49.6	−6.1	−3.3
4	10.6	14.3	10.7	−25.4
5	20.9	8.1	21.7	−0.4
6	11.8	41.5	26.2	44.8
7	37.2	50.4	20.9	−30.2
8	8.3	5.9	34.0	61.4
9	11.3	30.8	30.9	21.4
10	19.2	66.5	−7.0	6.8
11	29.0	54.5	−21.5	−17.9
Average	22.4	40.6	12.1	3.4
SD	13.4	23.1	20.7	31.8

Values are expressed as kcal/6 h.

### Reproducibility study

**Figure 1:**
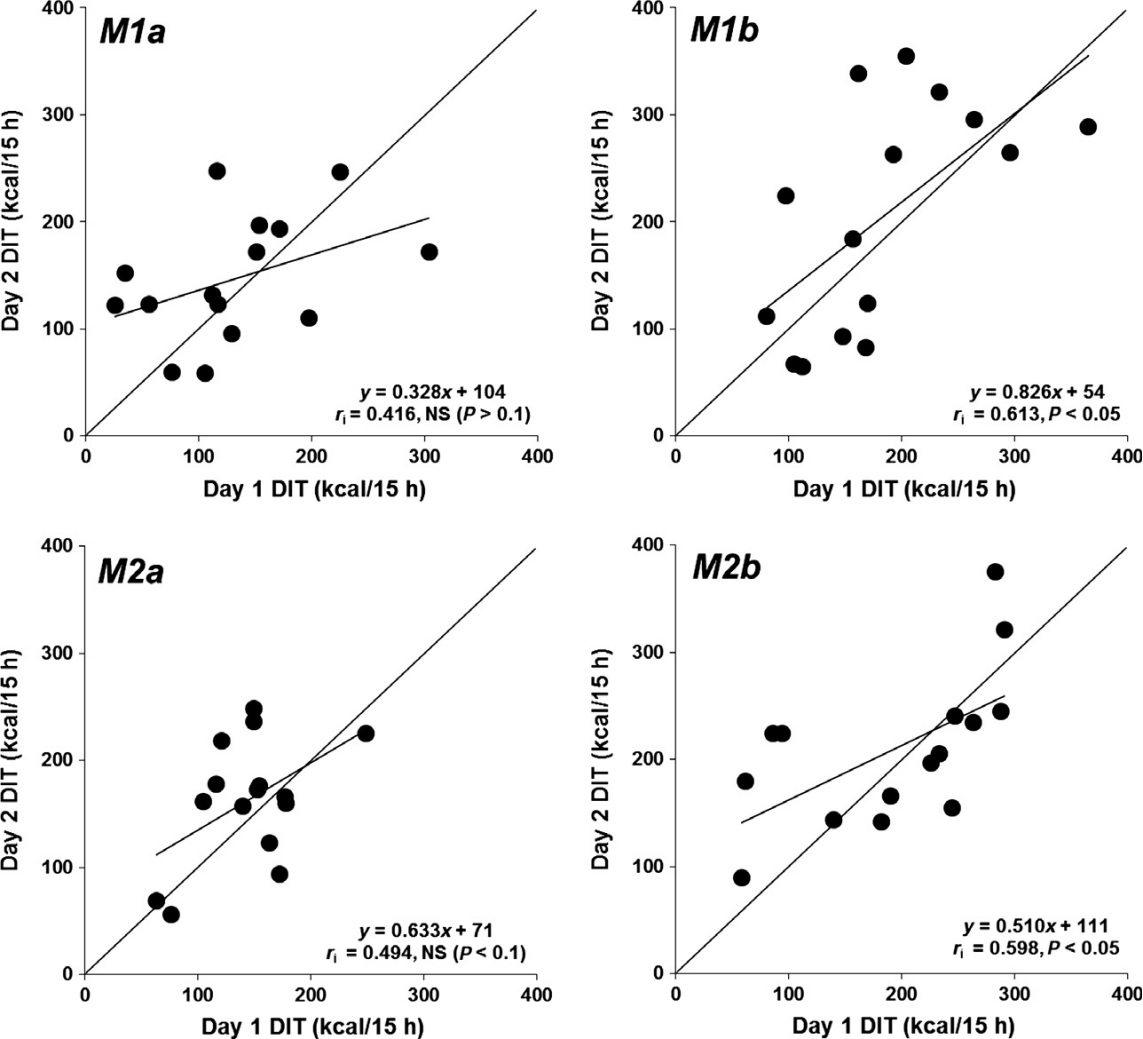
Scatter plot of 15 h DIT on *day 2* vs. *day 1* calculated using four methods using an indirect human calorimeter. *M1a*, novel NIHN method using a triaxial accelerometer; *M1b*, novel NIHN method using an infrared sensor system; *M2a*, Schutz's method using a triaxial accelerometer; *M2b*, Schutz's method using an infrared sensor system.

**Figure 2:**
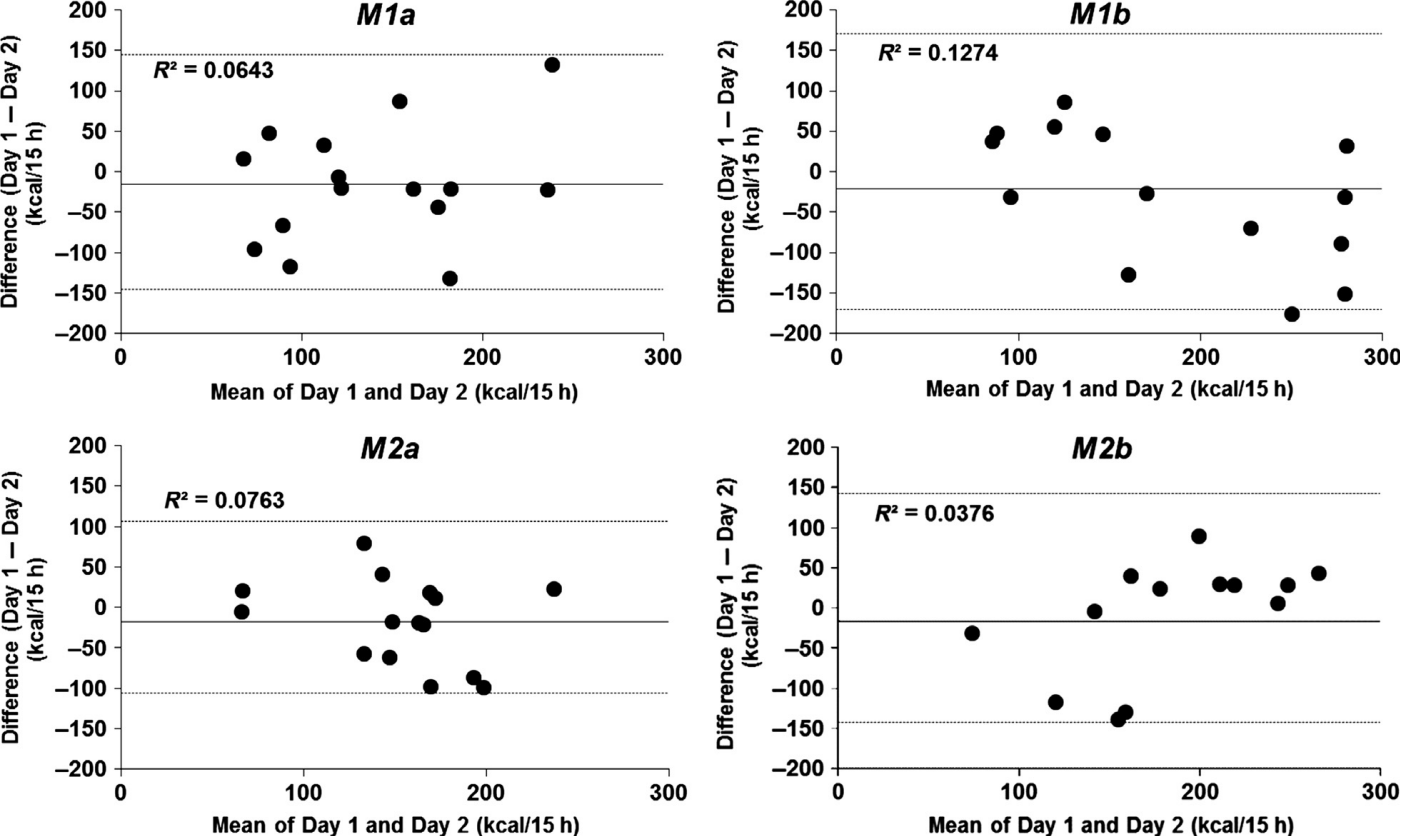
Bland-Altman plots of 15 h DIT calculated using four methods using an indirect human calorimeter. The mean values (mean of *day 1* and *day 2*) are plotted against the difference of the same two ratings. The interval mean ± 2 SD (mean ± CR [the coefficient of repeatability]) is shown by horizontal lines. *M1a*, novel NIHN method using a triaxial accelerometer; *M1b*, novel NIHN method using an infrared sensor system; *M2a*, Schutz's method using a triaxial accelerometer; *M2b*, Schutz's method using an infrared sensor system.

The reproducibility of 24 h TEE, sleeping metabolic rate (SMR), and 15 h DIT calculated using the four methods is shown in Table4. When duplicate measurements were obtained in 15 subjects, the mean 24 h TEE showed no significant difference between *days 1* and *2*. However, the SMR on *day 1* was significantly higher than that on *day 2*. The 24 h TEE and SMR were quite reproducible within-subjects, with day-to-day coefficient of variation (CV) of 2.4 and 3.0%, respectively. The mean values of DIT measured by the four methods accounted for 132–209 kcal/15 h representing 6.4–10.4% of the ingested calories (2008 ± 284 kcal) (Table4). There were no significant differences in the mean values of DIT (both kcal/15 h and %) between the two test days. Scatter plots of the regression equations and *r*_*i*_s are illustrated in Figure1. DITs calculated by the novel and traditional methods using a motion-detecting system (*M1b* and *M2b*) on *day 1* correlated closely with those on *day 2* (*M1b*: *r*_*i*_ = 0.613, *M2b*: *r*_*i*_ = 0.598, *P* < 0.05, respectively). In contrast, the DITs calculated using a triaxial accelerometer (*M1a* and *M2a*) on *day 1* did not correlate with those on *day 2* (*M1a*: *r*_*i*_ = 0.416, *P* = 0.123, *M1b*: *r*_*i*_ = 0.494, *P* = 0.061). However, there was no significant difference between the correlation coefficients of each of the four methods. In addition, Bland–Altman analysis did not show any bias in the variability in individual error scores of the four methods (Fig.2). The within-subject day-to-day CV for the calculated DITs was approximately 31–37% using the novel NIHN methods (*M1a* and *M1b*), and approximately 25–27% using Schutz's methods (*M2a* and *M2b*) (Table 4). There was no significant difference between the SD or % for each of the four methods.

**Table 4:** Comparison between the means of total energy expenditure, sleeping metabolic rate, and 15 h DIT between *days 1* and *2* of the experiment, calculated using the four methods.

	Day 1	Day 2	Difference	*P*	CV (%)
24-h TEE (kcal/day)	1837 ± 291	1808 ± 281	29 ± 58	NS	2.4
SMR (kcal/day)	1358 ± 227	1329 ± 207	30 ± 50	0.038	3.0
DIT (kcal/15 h)					
M1a	132 ± 74	147 ± 58	−15 ± 73	NS	36.4
M1b	183 ± 79	205 ± 106	−22 ± 85	NS	31.0
M2a	144 ± 45	163 ± 58	−18 ± 53	NS	25.0
M2b	192 ± 84	209 ± 72	−17 ± 71	NS	24.8
DIT (%)					
M1a	6.4 ± 3.2	7.2 ± 2.5	−0.8 ± 3.6	NS	36.6
M1b	9.0 ± 3.3	10.1 ± 4.9	−1.1 ± 4.2	NS	31.1
M2a	7.3 ± 2.4	8.3 ± 3.4	−0.9 ± 2.9	NS	27.0
M2b	9.6 ± 4.1	10.4 ± 3.1	−0.8 ± 3.7	NS	26.1

TEE, total energy expenditure; SMR, sleeping metabolic rate: the minimum EE during 3 consecutive hours of sleep; *M1a*, novel NIHN method using a triaxial accelerometer; *M1b*, novel NIHN method using an infrared sensor system; *M2a*, Schutz's method using a triaxial accelerometer; *M2b*, Schutz's method using an infrared sensor system; difference, value = Day 1–Day 2; NS, not significant.

The values are expressed as means ± SD.

## Discussion

Tataranni et al. (1995) determined DIT using Schutz's method and a radar system in a respiratory chamber, and showed that the method had very poor reproducibility (CV = 125%) and underestimated DIT compared with a method that evaluated the difference between feeding and fasting. In addition, Kumahara et al. (2011) demonstrated that DIT could not be determined by Schutz's method in 12% of subjects. In this study, DIT measured by Schutz's method showed negative values in over 30% of subjects. This was due to the *M2a* and *M2b* methods having negative effects in terms of accuracy in the regression equation (i.e., estimated BMRs were larger than the y-intercept at zero activity for EE in the reproducibility study). Moreover, the variation in DIT determined by the traditional method and a motion-detecting system (*M2b*) was larger compared with those obtained by the novel NIHN method using a triaxial accelerometer (*M1a*). These results confirm that Schutz's method underestimates DIT in a substantial number of cases, and indicates that it is necessary to overcome this shortcoming of the method. Previous studies have shown that one of the causes for underestimation of DIT by Schutz's method is that the single index of activity using the mean of the radar system data provides insufficient information for making assumptions on the magnitude of PA and EE. This leads to inaccurate calculation of DIT (Tataranni et al. 1995; Westerterp et al. 1999). In addition, subjects showed very little spontaneous PA in the chamber, due to its restrictive size. The fundamental assumption of covariation of EE with PA may be difficult to maintain as relatively small movements may cause variation in the differences in PA. Furthermore, because the regression equation in Schutz's method is computed from EEs and the PA index during the postprandial state, the slope and intercept of the equation may be affected by the timing of high-intensity PA in the chamber. For example, the slope of the regression equation in a subject performing high-intensity activities immediately after eating may be larger than the slope measured in another subject performing high-intensity activities hours after eating. As a few extreme points may influence the slope of the relationship, the intercept value of the regression line may be either over- or underestimated. This influence of extreme points may be one of the causes for differences in measurements of DIT. Westerterp et al. (1999) designed a light exercise protocol to ensure a greater range of EE and therefore eliminate the effect of erratic spontaneous PA. These authors suggested that including an exercise protocol is appropriate (Westerterp et al. 1999). Kumahara et al. (2011) reported a technique based on the traditional method that used an accelerometer and took the intensity of PA into account when computing DIT. This method achieved consistent and reliable results and also allowed for assessment of interindividual variability. Marino et al. (2002) reported goodness-of-fit achieved by the formula used to calculate DIT, and showed generally consistent and reliable DIT measurements. However, the real value of DIT still remains to be established.

To solve these problems, we focused on evaluating the relationship between EE and PA intensity, assessed in the fasting state using a triaxial accelerometer. The EE after an overnight fast includes only BMR and PAEE, and not DIT. Our studies showed that most of the intensity of spontaneous PA under free-living conditions in a respiratory chamber expressed as synthetic acceleration was < 30 mG, (data not shown). Our previous study showed that triaxial accelerometers estimate the intensity of sedentary, locomotive, and household activities accurately (Ohkawara et al. 2011). We therefore selected four PAs that best represented typical low-intensity PA in a respiratory chamber, such as resting in the sitting position, personal computer work, doing jigsaw puzzles, and folding laundry. This allowed a regression equation of EEs and PA intensity to be obtained for each individual. DIT was calculated as the difference between estimated EE and measured EE. We hypothesized that this novel technique that takes into account the relationship between PA intensity and EE after an overnight fast may help overcome the shortcomings of Schutz's technique, and may provide the real value and time-dependent change in DIT.

### Validity study

Our hypothesis was that DIT values calculated by our novel technique that used the regression equation for EE and PA index in the fasting state became zero kcal with small variations. However, the DIT values in *M1a* and *M1b* did not become zero, but were positive in all subjects. The mean DIT value calculated using the NIHN method and a triaxial accelerometer (*M1a*) was 22.4 kcal/6 h, with DIT values at 6 h being positive in all subjects. These results suggested that the novel NIHN method may overestimate DIT. During the experimental protocol in the chamber, the subjects were allowed to drink water ad libitum, although we did not record the amount of water ingested. Boschmann et al. (2003) reported that drinking 500 mL of water increased resting energy expenditure by 30%, and suggested that water-induced thermogenesis attributed to activation of the sympathetic nervous system. These authors also suggested that increasing daily water intake by 1.5 L would augment energy expenditure by approximately 48 kcal/day (200 kJ/d), that is, 2 kcal/h (Boschmann et al. 2003). In the present study we observed that DIT values calculated using the novel NIHN method (*M1a*) was 3.7 kcal/h (22.4 kcal/6 h). Because drinking a lot of water has the potential to affect energy expenditure, DIT values in the fasting state may not have reached zero kcal in our study. However, a previous study (Brown et al. 2006) that used different methodology, such as a ventilated hood system, suggested that drinking distilled water at room temperature did not increase energy expenditure, with the results inconsistent with the concept of water-induced thermogenesis. It is difficult to explain the discrepant results by these differences in methodology. We therefore need to further investigate the concept of water-induced thermogenesis, because it is possible that dissolved impurities or other substances in water may contribute to this process.

In addition, because there were large variations in the relationship between EE and PA intensity in an individual in the fasting state, we could not obtain a significant equation in some cases. The smaller amount of data used to obtain the equation in the novel NIHN method may be one cause of the negative results. In the calculation of DIT for 6 h, the novel NIHN method uses the regression equation obtained from 4 PAs, whereas Schutz's method uses the intercept of the regression equation of 12 data points (1 point per 30 min). Therefore, although we picked the good low-intensity PAs from the different kinds of PA in this pilot study, further discussion is required in order to calculate the regression equation between EE and PA intensity in the fasting state.

On the other hand, the variation in DIT should be distributed around zero, with large SD values indicating large inconsistent errors dependent on the subjects. In this study, the variation in DIT (*M1a*) was smaller than those obtained by the three other methods, although there was no significant difference in the homogeneity of variance between all four methods (*F* = 5.601, *P* = 0.071; adjusted by the Bonferroni method). These results indicate it is possible that the NIHN method and a triaxial accelerometer may be better for evaluating interindividual differences in DIT.

Regarding the resolution time for calculation of DIT by Schutz's method, we employed 30 min resolution based on the following comparison. We analyzed the data using 30 versus 10 versus 1 min resolutions in Schutz's method, and showed mean DIT calculated using a triaxial accelerometer (*M2a*) was 12.1 ± 20.7, 15.8 ± 13.8, and 38.4 ± 21.7 kcal/6 h, respectively. Mean DIT calculated using 30 or 10 min resolution was significantly lower than that calculated using 1 min resolution.

### Reproducibility study

This study confirms the findings of several other studies that EE can be measured in respiratory chambers with very high reproducibility (Ravussin et al. 1986; Astrup et al. 1990; Toubro et al. 1995; White et al. 1996). The 24 h TEE and SMR had a remarkably low CV (2.4 and 3.0%, respectively). We found that mean 24 h TEE was not significantly different between *days 1* and *2* in a chamber. However, the SMR on *day 2* was significantly lower than on *day 1* (*day 1,* 1358 ± 227 vs. day 2, 1329 ± 207 kcal/day, *P* = 0.038). The lower SMR on *day 2* was probably due to the subjects becoming used to living in a respiratory chamber and performing the experimental protocol, whereas the difference in SMR between 2 days was very small (about 30 kcal/day). These results demonstrated that EE explained by PA and DIT was similar on the two sessions.

Theoretically, in healthy subjects with a mixed diet, DIT represents about 10% of the total amount of energy ingested over 24 h (Van Zant 1992; Westerterp 2004). In fact, previous studies using a respiratory chamber showed that the mean relative value of DIT accounted for 5 to 15% of the total amount of energy ingested (Schutz et al. 1984; Ravussin et al. 1986; Minghelli et al. 1990; Tataranni et al. 1995; Verboeket-van de Venne et al. 1996; Marino et al. 2002; Lejeune et al. 2006; Smeets and Westerterp-Plantenga 2008; Kumahara et al. 2011). In this study, the mean DIT values measured using a triaxial accelerometer accounted for 132–163 kcal/15 h representing 6.4–8.3% of ingested calories (2008 ± 284 kcal) (Table 4, *M1a* and *M2a*). On the other hand, both mean absolute and relative values of DIT (183–209 kcal/15 h, 9.0–10.4%, respectively) measured using a motion-detecting system were higher than those measured by a triaxial accelerometer. Although the relative values of DIT (%) obtained by either one of the two devices to assess the PA index agreed closely with DIT reported by earlier studies, using a triaxial accelerometer to calculate DIT may provide lower DIT values compared with those calculated using a motion-detecting system.

In this study, there was no significant difference in mean DIT between the two test days (both kcal/15 h and %) (Table 4). Bland–Altman analysis did not indicate any bias in the variability in individual error scores of the four methods (Fig.2). However, DITs calculated using a triaxial accelerometer on *day 1* did not correlate with those measured on *day 2* (Fig.1, *M1a* and *M2a*). Although the cause of this lack of correlation between the two sessions is unknown, it is necessary to improve the degree of correlation in future studies. On the other hand, using Schutz's method, reported within-subject day-to-day CV for DIT is 43 to 48% (Ravussin et al. 1986; Tataranni et al. 1995), whereas interindividual variability in DIT is 58.0 to 151.7 (Ganpule et al. 2007; Ohkawara et al. 2011). In this study, within-subject variability in DIT (kcal/15 h and %) calculated using the novel NIHN method and a triaxial accelerometer (*M1a*) was 36.4 to 36.6%, which is slightly higher than the reproducibility of Schutz's method with an infrared sensor system (*M2b*; 24.8 to 26.1%, respectively). These results therefore suggest that our novel NIHN method that takes into account the relationship between PA intensity and EE after an overnight fast was not able to improve the reproducibility of Schutz's method.

Our investigation had several limitations. First, the sample sizes of our studies were relatively small. Further larger studies are therefore required to confirm the results and refine the novel NIHN method. Second, both techniques had methodological limitations. For example, Schutz's method is influenced directly by inaccuracies in SMR, whereas the novel NIHN method requires an individual calibration period during a fasted state and a specific activity protocol that may not be feasible for some studies. Finally, one possible explanation for the difference between the novel NIHN and Schutz's methods (Table 3) is the value of SMR. If these subjects did not completely achieve basal sleeping EE in the validation study, then this error could be carried over into DIT. However, we do not have any information on the state of sleeping for each subject in the validation study. Therefore, we need to take the variability in the sleeping and basal EE relationship into consideration.

In the future, there should be questions as to whether the following conditions improve this novel method; exercise, especially locomotive PA such as walking and jogging. The novel NIHN technique gives little regard to energy expenditure affected by locomotive PA. In addition, much of the spontaneous PA was not steady-state, so it is likely that this is also the case for PA during the DIT measurement periods. A PA protocol including both steady-state PA and nonsteady-state PA may therefore be more appropriate. We reported recently that sedentary activities could be discriminated from household and locomotive activities by accelerometer counts, and that locomotive activities could be distinguished from household activities using a cut-off determined as the ratio of unfiltered to filtered synthetic acceleration (Oshima et al. 2010; Ohkawara et al. 2011). These results suggest that the slope of the regression equation for the relationship between EE and synthetic acceleration during locomotive activities is different from the slope of the regression equation for household activities. Therefore, if the protocol for determining DIT in a respiratory chamber includes prolonged locomotive activities, our novel technique should add some locomotive activities to the special activities in the fasting state in the early morning, and derive the respective regression lines or curves of household and locomotive activity before calculating DIT. By this method it may be possible to accurately assess DIT.

**Figure 3:**
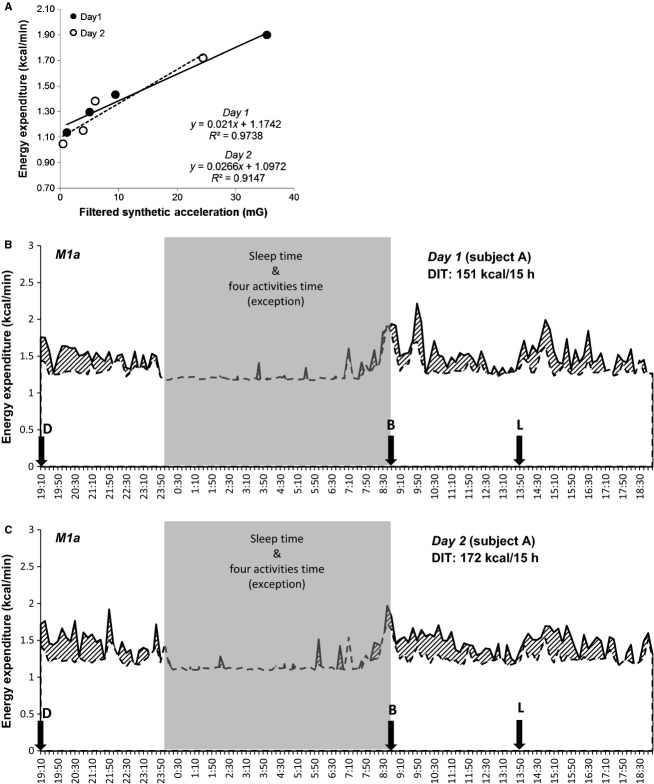
Relationship between energy expenditure and specific activity, and energy expenditure over 24 h in one subject. (A): relationship between energy expenditure and four specific activities during two sessions. (B) and (C): energy expenditure over 24 h during two sessions. The upper solid line represents the total energy expenditure averaged over 10 min periods. The dotted line represents the estimated energy expenditure including resting energy expenditure and physical activity-related energy expenditure. The dashed area represents the value of diet-induced thermogenesis. The following letters represent meal start times: D, dinner; B, breakfast; L, lunch.

In conclusion, our novel NIHN technique that considers the relationship between the intensity of PA and EE after an overnight fast did not have superior reproducibility compared with Schutz's technique. On the other hand, we were able to calculate absolute values of DIT and PA-related EE separately from the time course. This novel NIHN technique may, however, overestimate DIT and therefore it is necessary to perform some special activities for more than an hour in the fasting state in the early morning (Fig.3). Our results raise the possibility that the NIHN method using a triaxial accelerometer may be better for evaluating interindividual differences in DIT. It is also possible that our novel method in the fasting state may evaluate special energy expenditure, for example, “excess postexercise oxygen consumption” (EPOC) in the same way as this study. From the viewpoint of issues of obesity and prevention of lifestyle-related disease, it may be necessary to observe intra and interindividual differences in DIT variations associated with meals and time of food intake. This study using a respiratory chamber should contribute to determining the connection between components of daily energy expenditure and physiological and biochemical indices.
